# Child Abuse, Misdiagnosed by an Expertise Center—Part II—Misuse of Bayes’ Theorem

**DOI:** 10.3390/children10050843

**Published:** 2023-05-06

**Authors:** Martin J. C. van Gemert, Aeilko H. Zwinderman, Peter J. van Koppen, H. A. Martino Neumann, Marianne Vlaming

**Affiliations:** 1 Department of Biomedical Engineering & Physics, Amsterdam University Medical Centers, Location AMC, University of Amsterdam, Meibergdreef 9, 1105 AZ Amsterdam, The Netherlands; 2 Department of Clinical Epidemiology & Bio-Statistics, Amsterdam University Medical Centers, Location AMC, University of Amsterdam, 1105 AZ Amsterdam, The Netherlands; 3 Department of Criminal Law and Criminology, Faculty of Law, VU University, 1081 HV Amsterdam, The Netherlands; 4 ZBC-Multicare, 1217 AB Hilversum, The Netherlands; 5 Private Practice, Criminal Psychology and Law, 6986 CL Angerlo, The Netherlands

**Keywords:** infant bruises, rib fractures, Bayes’ theorem, infant physical abuse probability, unfounded likelihood ratios, misdiagnosed child abuse, hypermobility-type Ehlers-Danlos-Syndrome (hEDS)

## Abstract

A newborn girl had, from two weeks on, small bruises on varying body locations, but not on her chest. Her Armenian grandmother easily bruised, too. Her mother was diagnosed with hypermobility-type Ehlers-Danlos-Syndrome (hEDS), an autosomal dominant connective tissue disorder, with a 50% inheritance probability. Referral to a University Medical Center located “*Dutch Expertise Center for Child Abuse*” resulted (prior to consultation) in physical abuse suspicion. Protocol-based skeletal X-rays showed three healed, asymptomatic rib fractures. A protocol-based Bayesian likelihood ratio guesstimation gave 10–100, erroneously used to suggest a 10–100 times likelier non-accidental-than-accidental cause. Foster care placement followed, even in a secret home, where she also bruised, suggesting hEDS inheritance. Correct non-accidental/accidental Bayes’ probability of symptoms is (likelihood ratio) × (physical abuse incidence). From the literature, we derived an infant abuse incidence between about ≈0.0009 and ≈0.0026 and a likelihood ratio of <5 for bruises. For rib fractures, we used a zero likelihood ratio, arguing their cause was birth trauma from the extra delivery pressure on the chest, combined with fragile bones as the daughter of an hEDS-mother. We thus derived a negligible abuse/accidental probability between <5 × 0.0009 <0.005 and <5 × 0.0026 <0.013. The small abuse incidence implies that correctly using Bayes’ theorem will also miss true infant physical abuse cases. Curiously, because likelihood ratios assess how more often symptoms develop if abuse did occur versus non-abuse, Bayes’ theorem then implies a 100% infant abuse incidence (unwittingly) used by LECK. In conclusion, probabilities should never replace differential diagnostic procedures, the accepted medical method of care. Well-known from literature, supported by the present case, is that (child abuse pediatrics) physicians, child protection workers, and judges were unlikely to understand Bayesian statistics. Its use without statistics consultation should therefore not have occurred. Thus, Bayesian statistics, and certainly (misused) likelihood ratios, should never be applied in cases of physical child abuse suspicion. Finally, parental innocence follows from clarifying what could have caused the girl’s bruises (inherited hEDS), and rib fractures (birth trauma from fragile bones).

## 1. Introduction

In Part I [1], we described an Armenian (father)-Dutch girl, uncomplicated term birth, who developed from about two weeks onwards small bruises on varying body locations, but not on her chest. Her paternal grandmother easily bruised too. At 2 months of age, the girl was referred to a “Landelijk Expertise Centrum Kindermishandeling”: “LECK” (“Dutch Expertise Center for Child Abuse”), located at a university medical center, where LECK pediatricians immediately, even before consultation, suspected child abuse. The threat of child removal made the parents comply with the hospital’s child abuse investigations, where three healed, not dislocated, anterolateral asymptomatic, left-side rib fractures were found. The LECK child abuse program includes the use of likelihood ratios [2], part of Bayesian statistics (see Section 2.1. below), to come to a conclusion.

Without mathematical details, they guesstimated a likelihood ratio of 10–100 and concluded (incorrectly, see below) that abuse was therefore 10–100 times more likely than an accidental cause. A child protection worker reported suspicion of aggravated assault to the police, which triggered the roller coaster of prosecutions and involvement of three Dutch child protection organizations. For the girl and her 20 months older brother, placement for 8 months in foster care, also in a secret home, resulted. The case required five court procedures (four civil and one criminal). The legal and moral aspects of this case will be dealt with in the forthcoming Part III. The children returned home again after the mother was diagnosed with hypermobility-type Ehlers-Danlos Syndrome (hEDS) [1], and when a pediatric and radiologic second opinion confirmed that the mother’s hEDS could have affected the girl’s bruises, that her rib fractures could well have been developed perinatally, while the observed widened metaphyses suggested that an underlying bone disease could well be possible.

Ehlers-Danlos Syndrome (EDS) is a heterogeneous group of 14 autosomal dominant connective tissue disorders, part of the spectrum of inherited metabolic diseases, caused by abnormal collagen synthesis that affects the skin and various other organs [3,4]. The most prevalent type is hEDS. Children have a 50% probability of acquiring this disorder too [3,4]. It manifests itself clinically with soft and hyper-elastic skin, abnormal wound healing, easy bruising, and joint hypermobility [5]. Its incidence is not precisely known, reported between about 1:5000 [6] and 1:500 [7], see also the review by Malfait et al. [8]. In children, hEDS is poorly recognized from a lack of awareness by physicians and child protection workers, frequently leading to misdiagnosed child abuse with temporary (as in our case), or even permanent foster care placement of the child.

In this paper, we address Bayes’ theorem, named after Thomas Bayes, an 18th-century English statistician, philosopher, and Presbyterian minister [9]. His method reflects updating the probability of a hypothesis (here, child abuse caused the symptoms), as more evidence about the symptoms becomes available (called a likelihood ratio), i.e., how more often symptoms develop if child abuse indeed occurred compared to the alternative non-abuse probability. The updated outcome is the relative probability of the hypothesis being true if the symptoms are present (called posterior odds). The prior odds are the relative physical abuse incidence here. Bayes’ theorem has found application in a wide range of activities, including science, engineering, philosophy, medicine, sport, and law (see, e.g., Wikipedia’s chapter [10]).

First, the four scientific aims of this paper are to explain Bayes’ theorem, present the correct analysis of the case, derive an estimated incidence of physical abuse in Dutch infants, and show that the probability of a non-accidental versus an accidental cause of the symptoms is extremely small (≈<0.013). Second, to show that LECK’s guesstimated likelihood ratio of 10–100 is unrelated to their conclusion that the symptoms are thus 10–100 times more likely non-accidental than accidental. Third, to provide the scientific bone-fragility-related-arguments for parental innocence. Additionally, to explain why application of Bayes’ theorem should never be used in cases of physical child abuse suspicion. Finally, to suggest research directions for follow-up investigations.

## 2. Methods

### 2.1. Bayes’ Theorem

Bayes’ statistics uses (here) the probability (P) that the infant’s bruises and rib fractures occur due to “*Abuse*” as the primary cause, and the probability that they occur due to an “*Alternative*” cause, or, using the notation $P\left(A|B\right)$, i.e., the probability that A is observed (the infant’s bruises and rib fractures) given the assumption of B (the child has actually been physically abused or there were alternative causes) gives

(1) $$P\left(\mathrm{BruiRibFr}|\mathrm{Abuse}\right)\;\mathrm{and}\;P\left(\mathrm{BruiRibFr}|\mathrm{Alternative}\right)$$

The likelihood ratio is then defined as the ratio of these two probabilities

(2a) $$\mathrm{LikelihoodRatio}\left(\mathrm{BruiRibFr}\right)=\frac{P\left(\mathrm{BruiRibFr}|\mathrm{Abuse}\right)}{P\left(\mathrm{BruiRibFr}|\mathrm{Accidental}\right)}$$

(2b) $$\mathrm{Likelihood}\;\mathrm{Ratio}=\frac{\mathrm{Probability}\;\mathrm{that}\;\mathrm{the}\;\mathrm{observed}\;\mathrm{symptoms}\;\mathrm{develop}\;\mathrm{if}\;\mathrm{physical}\;\mathrm{abuse}\;\mathrm{occurred}}{\mathrm{Probability}\;\mathrm{hat}\;\mathrm{the}\;\mathrm{observed}\;\mathrm{symptoms}\;\mathrm{develop}\;\mathrm{if}\;\mathrm{accidental}\;\mathrm{causes}\;\mathrm{occurred}}$$

Thus, what a likelihood ratio does *not* deliver is the probability that abuse causes the observed symptoms, see Equation (4) below.

Thus far, the argument is standard science, as for instance exemplified by Karl Popper [11]. It expresses that a hypothesis is always tested against at least one alternative hypothesis. Bayes used that notion to give a statistical model in which the relative subjective strength of two hypotheses can be adjusted to the information introduced by new evidence. It starts with prior odds.

The prior odds of abuse is the ratio of the general probability of physical abuse, here in young infants, compared to accidental causes, where these probabilities sum up to 1, or

(3) $$\frac{P\left(\mathrm{Abuse}\right)}{P\left(\mathrm{Accidental}\right)}=\frac{P\left(\mathrm{Abuse}\right)}{1-P\left(\mathrm{Abuse}\right)}$$

The posterior odds is then given by Bayes’ theorem

(4a) Posterior Odds=Likelihood Ratio×Prior Odds

(4b) $$\frac{P\left(\mathrm{Abuse}|\mathrm{BruiRibFr}\right)}{P\left(\mathrm{Accidental}|\mathrm{BruiRibFr}\right)}=\frac{P\left(\mathrm{BruiRibFr}|\mathrm{Abuse}\right)}{P\left(\mathrm{BruiRibFr}|\mathrm{Accidental}\right)}\times \frac{P\left(\mathrm{Abuse}\right)}{P\left(\mathrm{Accidental}\right)}$$

The starting probability that the observed bruises and rib fractures are indeed caused by abuse =
how more often bruises and rib fractures develop when abuse did occur versus accidental causes × 
the probability of physical abuse versus accidental causes (4c)

### 2.2. Alternative Description of Bayes’ Theorem

In an alternative way, Bayesian statistics combines the two probabilities $P\left(\mathrm{BruiRibFr}|\mathrm{Abuse}\right)$ and $P\left(\mathrm{BruiRibFr}|\mathrm{Alternative}\right)$ of Equation (1) to express the posterior probability that abuse occurred, given that the observed bruises and rib fractures can result from abuse as well as accidental causes, or

(5) $$P\left(\mathrm{Abuse}|\mathrm{BruiRibFr}\right)=\frac{P\left(\mathrm{BruiRibFr}|\mathrm{Abuse}\right)*P\left(\mathrm{Abuse}\right)}{P\left(\mathrm{BruiRibFr}|\mathrm{Abuse}\right)*P\left(\mathrm{Abuse}\right)+P\left(\mathrm{BruiRibFr}|\mathrm{Accidental}\right)*P\left(\mathrm{Accidental}\right)}$$

(6) $$P\left(\mathrm{Accidental}|\mathrm{BruiRibFr}\right)=\frac{P\left(\mathrm{BruiRibFr}|\mathrm{Accidental}\right)*P\left(\mathrm{Accidental}\right)}{P\left(\mathrm{BruiRibFr}|\mathrm{Accidental}\right)*P\left(\mathrm{Accidental}\right)+P\left(\mathrm{BruiRibFr}|\mathrm{Abuse}\right)*P\left(\mathrm{Abuse}\right)}$$

Then, dividing Equation (5) by Equation (6), gives Equation (4b) again.

### 2.3. Estimated Incidence of Physical Abuse in Dutch Infants

Based on the US Department of Health & Human Services, Children’s Bureau: 2020, the USA incidence of abuse in children under 1 year in 2020 has been estimated as about 2.51% (their Tables 3–5, page 41) of which an estimated 16.5% is due to physical abuse (page 11). We used their *physical abuse* incidence, or $P\left(\mathrm{PhysicalAbuse}\right)$ ≈ 0.165 × 0.0251 ≈ 0.00414. However, in WebTable 2 of the Supplement to Gilbert et al. [12], the incidence of “*Maltreatment syndrome or assault*” is in Sweden a factor of 11.5/54.4 ≈ 0.211 and in the UK a factor of 34.6/54.4 ≈ 0.636 smaller than in the USA. Because child abuse in the Netherlands is more likely comparable to those in Sweden and the UK than the USA, we will use for our case that the physical abuse incidence is between

(7a) $$\frac{P\left(\mathrm{PhysicalAbuse}\right)}{P\left(\mathrm{Accidental}\right)}\approx 0.211\times 0.00414\approx 0.0009$$

(7b) $$\frac{P\left(\mathrm{PhysicalAbuse}\right)}{P\left(\mathrm{Accidental}\right)}\approx 0.636\times 0.00414\approx 0.0026$$

## 3. Results

We found no evidence that the LECK pediatricians attempted to derive a one likelihood ratio, so separate likelihood ratios must have been used. Yet, their only likelihood ratio estimate was 10–100. Here, we apply Bayes’ theorem separately for the bruises (Section 3.1) and for the rib fractures (Section 3.2).

### 3.1. Bayes’ Theorem for the Bruises

We have to evaluate

(8) $$\frac{P\left(\mathrm{Abuse}|\mathrm{Brui}\right)}{P\left(\mathrm{Accidental}|\mathrm{Brui}\right)}=\frac{P\left(\mathrm{Brui}|\mathrm{Abuse}\right)}{P\left(\mathrm{Brui}|\mathrm{Accidental}\right)}\times \frac{P\left(\mathrm{Abuse}\right)}{P\left(\mathrm{Accidental}\right)}$$

For bruises, LECK used the work of Kemp et al. [13], particularly their Table 3, where the percentage of infants with at least one bruise on 17 body locations was mentioned, for 107 physical abuse diagnosed babies and 30 physical abuse excluded babies. Kemp’s Figure 1 explains that in the 519 included children <6 years, physical abuse was not only based on witnessed or admitted abuse (24 cases), but also on much larger numbers of alleged abuse (52 abuse of 62 children), unexplained injury (149 abuse of 170) and concerning history (101 of 200). Given the possibility of false positive cases due to selection bias and misdiagnosis, e.g., EDS and osteogenesis imperfecta cases were not included, we question the accuracy of the 107 abused and the 30 non-abused infants. However, we can only compare the body locations in Kemp’s Table 3 where bruising occurred in the abused and the non-abused diagnosed infants but know that these ratios are in reality too large. The largest abuse/accidental ratio of five was on the front side of the infant trunk; all other locations had a smaller value. Thus, for convenience, we used

(9) $$\mathrm{LikelihoodRatio}\left(\mathrm{Bruises}\right)<5$$

Then, respectively for the Swedish (Equation (10a)) and the UK (Equation (10b)) values

(10a) $$\frac{P\left(\mathrm{Abuse}|\mathrm{Brui}\right)}{P\left(\mathrm{Accidental}|\mathrm{Brui}\right)}=\frac{P\left(\mathrm{Brui}|\mathrm{Abuse}\right)}{P\left(\mathrm{Brui}|\mathrm{Accidental}\right)}\times \frac{P\left(\mathrm{Abuse}\right)}{P\left(\mathrm{Accidental}\right)}<5\times 0.0009<0.005$$

(10b) $$\frac{P\left(\mathrm{Abuse}|\mathrm{Brui}\right)}{P\left(\mathrm{Accidental}|\mathrm{Brui}\right)}=\frac{P\left(\mathrm{Brui}|\mathrm{Abuse}\right)}{P\left(\mathrm{Brui}|\mathrm{Accidental}\right)}\times \frac{P\left(\mathrm{Abuse}\right)}{P\left(\mathrm{Accidental}\right)}<5\times 0.0026<0.013$$

which shows a virtually negligible range of <0.5% to <1.3% predicted Bayesian posterior odds that the bruises in our case were indeed inflicted by physical abuse.

### 3.2. Bayes’ Theorem for the Rib Fractures

The likelihood ratio for infant rib fractures is defined as

(11) $$\mathrm{LikelihoodRatio}\left(\mathrm{RibFr}\right)=\frac{P\left(\mathrm{RibFr}|\mathrm{Abuse}\right)}{P\left(\mathrm{RibFr}|\mathrm{Accidental}\right)}$$

The literature suggests, e.g., [14], that compressive rib fractures are seldom accompanied by bruising, albeit that the effects of metabolic diseases were not accounted for, see, e.g., [15]. Thus, under normal conditions, observing that bruises were never found on the girl’s chest would not imply that the rib fractures and bruises were caused by different mechanisms. However, in Appendix A, we argue that both non-EDS and hEDS fetuses in a pregnant hEDS mother develop reduced bone strength, most severely in hEDS fetuses. Here, the fact that the girl kept making small bruises, also in the secret foster home [1], strongly suggests that she also suffered from hEDS and that her rib fractures were not abuse-related. In addition, the girl’s second-opinion-based radiologic observation of widened metaphyses made the radiologist suggest the possibility of an underlying bone disease.

We thus postulated that the girl’s rib fractures could well have been caused by the increased pressure during delivery, or perhaps already during pregnancy, which cannot be excluded because rib fractures are not routinely seen in utero by ultrasonography (*Michael G. Ross, personal communication to MJCvG*). These arguments explain the girl’s symptoms and thus prove the innocence of her parents.

An estimate of Equation (11) can be made by assuming that, if the girl’s rib fractures would have been caused by physical abuse, she would have had bruises on the underlying chest skin locations. Because this was not the case, we assumed a zero abuse probability for the rib fractures here, so

(12) $$\mathrm{LikelihoodRatio}\left(\mathrm{RibFr}\right)=\frac{P\left(\mathrm{RibFr}|\mathrm{Abuse}\right)}{P\left(\mathrm{RibFr}|\mathrm{Accidental}\right)}=0$$

(13) $$\frac{P\left(\mathrm{Abuse}|\mathrm{RibFr}\right)}{P\left(\mathrm{Accidental}|\mathrm{RibFr}\right)}=\frac{P\left(\mathrm{RibFr}|\mathrm{Abuse}\right)}{P\left(\mathrm{RibFr}|\mathrm{Accidental}\right)}\times \frac{P\left(\mathrm{Abuse}\right)}{P\left(\mathrm{Accidental}\right)}=0$$

Summarizing, combining Equations (10) and (13) gives that the abuse probability of the bruises and rib fractures is an estimated range between <0.005 and <0.013 posterior odds, thus negligible, and a factor of between 2000–20,000, respectively between 1000–10,000 smaller than LECK ‘s 10–100 guesstimation. Finally, even if the true likelihood ratio would have been 100, the 0.26% largest estimated infant relative abuse incidence would still make the probability of physical abuse compared to an accidental cause as small as about 0.0026 × 100 = 0.26, so still statistically negligible.

## 4. Discussion

### 4.1. Introductory Remarks

This paper is Part II of three related articles on a case of misdiagnosed physical child abuse by LECK pediatricians with serious consequences of the wrongly accused parents and their two children. Part I [2] describes the medico-social aspects, including why both children were kept in foster care for 2.5 months while under legal family supervision order for 8 months, and future Part III will focus on the legal and moral aspects. When we started drafting, it became rapidly clear that one single manuscript would become unreadable. Particularly, the complexity of Bayesian statistics for non-statisticians required the present separate paper. In Part I, we explained that LECK offers healthcare professionals an anonymous advice without requesting permission from caregivers and without observing the child themselves. LECK is not further involved in the cases. LECK is part of three Dutch University Hospitals, and in our opinion, it cannot be excluded that more will become involved. In 2018, LECK received 229 advisory requests [2].

### 4.2. On Bayesian Statistics

Essentially is here that Bayesian likelihood ratios are just one part of Bayes’ theorem and the full relation, Equation (4), including the general probability of physical abuse in young infants, Equation (7), should have been used by LECK. Because likelihood ratios just express how more frequent symptoms develop when abuse actually did occur versus accidental causes, Equation (2), it is obvious that likelihood ratios do *not* predict the probability that the observed symptoms followed non-accidentally. Nevertheless, that circular argument was used by LECK ‘s child abuse pediatricians, at best demonstrating their limited understanding of Bayes’ theorem. Support for that assumption is that their analysis in fact implies the use of a 100% infant physical abuse incidence.

A famous example of misused statistics (by a famous pediatrician) was the double sudden infant death syndrome in the family of Sally Cark [16]. After being sentenced to life in prison, the British *Royal Statistical Society* issued a public statement expressing its concern at the “*misuse of statistics in the courts*”. For the second appeal, a prosecution forensic pathologist commented: “*It was clear that sound medical principles were abandoned in favour of over-simplification, over-interpretation, exclusion of relevant data and, in several instances, the imagining of non-existent findings*”. Clark was released from prison after having served more than 3 years of her sentence.

Literature exists (e.g., [17,18]) on child abuse suspicion, misdiagnosed by child abuse pediatricians from misused statistical probabilities, which provided them with false certainties, as in our case. Some typical statements are as follows. From [17]: “*When probabilities and evidence based science are studied and applied, deep flaws in the fund of knowledge of child abuse pediatrics have been exposed. There is an emerging reality that the collective suffering of falsely accused families may dwarf the horrific impacts associated with real abuse.*” “*A false accusation of child abuse is child abuse.*” From [18]: “*Child abuse physicians routinely ignore the base rate* (abuse incidence, Equation (4)), as in this case.

However, the Bayesian approach does have an important place in medicine, particularly in predicting whether a new medicine/therapy may do better in treating disease than the standard, or how effective a therapy is. Then, the likelihood ratio becomes the ratio of the sensitivity (probability that a test gives the correct diagnosis) and the specificity (probability that a test fails to give the correct diagnosis), and each new case contributes to a more precise likelihood ratio. Thus,

(14) $$P\left(\mathrm{Test}\;\mathrm{Predicts}\;\mathrm{Disease}\right)=\frac{P\left(\mathrm{Test}\;\mathrm{is}\;\mathrm{Correct}\right)}{P\left(\mathrm{Test}\;\mathrm{is}\;\mathrm{Incorrect}\right)}\times P\left(\mathrm{disease}\right)$$

An example, presented by Woertman et al. [19], is whether Simvastatin and Atorvastatin give the same or different lipid reduction values in hypercholesterolemia patients. Atorvastatin turned out to be the better medicine. Another example is the Statistical Note on “*Diagnostic test 4: likelihood ratios*” [20], which was applied to study the value of a smoking history in diagnosing obstructive airway disease.

Finally, parental innocence follows by giving an explication of the girl’s bruises and rib fractures. First, the likelihood that the girl is hEDS-affected explains her bruising prevalence, supported by the large bruise on her leg in the secret foster care home [1]. Second, as (hEDS) daughter of an hEDS mother, she developed bone fragility (Appendix A), which likely caused her rib fractures from birth trauma due to the extra pressure exerted on her ribs during delivery.

### 4.3. Why Bayes’ Theorem Should Never Be Applied in Cases of Physical Child Abuse Suspicion

First, we recall that Bayesian likelihood ratios do *not* provide the probability that the symptoms did indeed occur from child abuse. Nevertheless, their values can be very large, and physicians may therefore not feel a further need to keep searching for evidence of an accidental cause, which mechanism likely contributes to the deep flaws observed in child abuse pediatrics [17,18]. Second, when correctly applied, i.e., Equation (4), the general probability of physical abuse has to be included. For Dutch infants, we derived a very small estimate of between ≈0.09% and ≈0.26%, Equation (7). Further, Gabaeff ([17], his paragraph 3.4), even derived a 0.003% incidence of “*prosecutable abuse for any parent in the USA*”. In other words, the (correct) use of Bayes’ theorem will (virtually) always miss true cases of tragic child abuse. Third, the use of probabilities to decide whether symptoms are due to physical child abuse or not should never replace differential diagnostic procedures, the generally accepted medical method of care. Fourth, the complexity of Bayesian statistics makes it obvious that (child abuse pediatrics) physicians, child protection employees and judges have little or no idea what Bayesian statistics are and what they do [21]. Combined with the fact that the status of LECK made their statements uncritically accepted by all involved, allows LECK to predict whatever they want, even if nonsense, by lack of a controlling mechanism. In future cases, we suggest that consulting an expert statistician in clinical practice and also in legal court procedures, could prevent this unwanted situation, which likely increases the number of unfounded abuse suspicion cases, including temporary or even permanent foster care placement. In child abuse cases where Bayesian statistics could play a role, e.g., as explained by Tadei et al. [22] (next paragraph), it is obvious that the correct relation should be used rather than the misused version of LECK. Then, it would make no sense anymore to use Bayes’ theorem for physical child abuse assessments, which may suggest the obsoleteness of LECK’s approach and more hope for caregivers who might otherwise become suspected of physical child abuse.

### 4.4. Suggestion for Research Directions for Follow-Up Investigations

Our first suggestion is to collect and analyze as many as possible physical child abuse cases similar to the present one to identify informative background information that may support clinical decision-making. An example is described by Tadei et al. [21] on the assessment of child sexual abuse allegations. These authors studied 903 demographic and socioeconomic variables from over 11,000 Finnish children and identified 42 features related to sexual abuse as summarized in Appendix A. They then included these 42 features in a complex computer program of Bayesian logic to calculate the probability of sexual abuse.

The second suggestion is to study in more detail the occurrence of bruising and asymptomatic rib fractures in newborns of hEDS and osteogenesis imperfecta mothers so that pediatricians and pediatric radiologists become more aware of this problem.

The third suggestion is to further study the occurrence of rib fractures in newborns, which are virtually always asymptomatic; hence, its incidence is unknown, thus the likelihood ratio as well, so the incidence of physical abuse as a possible cause of rib fractures is also currently unknown. Yet, multiple publications claim that their incidence can virtually only come from physical abuse (see Appendix A below).

## Data Availability

The data is contained within the article.

## Appendix A. Bone Fragility and Prenatal/Neonatal Rib Fractures

Bishop et al. [23] reviewed the pathophysiological aspects of bone disease in infants presenting with unexplained fractures and discussed disease processes that result in bone fragility. They particularly focused on metabolic bone disease of prematurity and osteogenesis imperfecta but not on hEDS. Further, Charoenngam et al. [24] published an extensive review on the diagnosis and management of pediatric metabolic bone diseases associated with skeletal fragility. Recently, Holick et al. [25] described fetal fractures in a 32 weeks pregnant hEDS-diagnosed mother, where the CCDC134 gene was determined in the infant. *From the Online Mendelian Inheritance in Man* (*OMIM*) database, this implies #619795 osteogenesis imperfecta 22.

A well-known fact in pediatric pathology (Peter G.J. Nikkels, personal communication to MJCvG) is that neonatal bone strength, including bone fragility, depends on the level of fetal movements during the 2nd and 3rd trimesters of pregnancy. The resulting strain on the various skeletal bones caused by these movements was considered the most important mechanism that controls fetal/infant bone strength, e.g., [26,27]. Support for that statement comes from case series that describe bone fragility and fractures from fetal immobility due to congenital neuromuscular disorders [28,29,30,31,32,33], intrauterine confinement of twins [34], and restricted fetal mobility due to a short umbilical cord [35]. What is also needed here is a normal functioning placenta.

If the placenta is malfunctioning, for example in cases of a serious chronic histiocytic intervillositis or a massive perivillous fibrin deposition, fragile bones and fractures have also been described [36,37].

Bone strain can occur from forces that act on the skeleton when the fetus and/or extremity strikes against the uterine wall, from muscle contractions, and as drag forces from movements in the amniotic fluid. Fetal movement itself, e.g., whole-body, trunk, limbs, breathing, hiccups, and stretching movements, can also create a strain on bones [27]. Further, based on Rauch and Schoenau [38,39], Miller [27] mentioned that changes in bone density and bone geometry in the first 6 months of life cause a three times greater bone strength at 6 months compared to that at birth, and fewer fractures beyond 6 months than before. Lower bone mineral density and fractures also occur in older EDS patients [40,41,42,43,44,45]. However, it is well possible that the cause here is the difference in food intake between EDS and non-EDS-affected individuals [46].

A possible but perhaps speculative explanation comes from Miller [27], who stated that joint hypermobility in the parent(s) and/or the fetus is another risk factor for creating bone fragility and even accidental fetal and neonatal fractures. Following Miller [27], an hEDS fetus will have softer skin, and hyper-elastic tendons and ligaments, compared with non-hEDS fetuses, and consequently, also more flexible extremities. Thus, hEDS fetuses could possibly have a reduced strain on their bones. Further, the uterus of an hEDS mother is likely softer and more elastic than normal because the uterus includes layers of elastin and collagen around the smooth muscle layer. Hence, a normal and an hEDS fetus in such an hEDS-related uterus may experience a reduced strain in their bones; the most severe form obviously is in an hEDS fetus in the uterus of an hEDS pregnant mother, as was the likely scenario in this case.

Further, neonatal fractures can also develop due to prematurity and birth trauma [47,48]. Birth trauma due to bone fragility was considered the cause of the girl’s rib fractures here.

In a bizarre comment, Brown et al. [49], representing the (American) “*Society for Pediatric Radiology (SPR) Child Abuse Committee*”, stated that it was “*grossly irresponsible*” and could present a “*grave public health risk*” that Miller et al. [26] reported their findings on genetic causes of accidental bone fragility and fractures. Miller’s strong and convincing reply followed [50]. Further, Brown et al. [49] referred to Strause [51], as “*an example of the evidence based medical literature*”, who invented the term (child abuse) “*denialists*”. Miller et al. [50] commented: “*We note that some members of the SPR have accused those who disagree with their approach to diagnosing child abuse of being ‘denialists,’ that is, of denying that child abuse exists. This is incorrect. We believe that child abuse exists*”. Additionally, Pfeifer [52] used this term but without referring to Strause.

These publications [49,50,51,52] demonstrate the serious disagreement that currently exists worldwide between child abuse specialized physicians and physicians who explore pathophysiologic ways that could significantly reduce current unjustified child abuse diagnoses. Brown et al. [49] did not reference publications showing that genetically affected infants, e.g., osteogenesis imperfecta and hypermobility spectrum/EDS syndrome, are frequently misdiagnosed as child abuse [12,53,54], and neither to recent population-based registry studies of 1,855,267 infants by Professor Ulf Högberg, Uppsala, Sweden (e.g., [15]).
